# 

**Published:** 1895-01-12

**Authors:** 


**Notices of Births, Deaths, and Marriages** are inserted in this column at a charge of 2s. 6d. for an announcement not exceeding four lines.

**Notice to Advertisers and Subscribers.**

All Advertisements, Orders for Copies of The Hospital, and Business Communications should be addressed to **The Manager, NOT TO THE EDITOR,** The Hospital, 428, Strand, London, W.C.

The Cost of Subscription, post free, is as follows:—Per week, 2½d.; per quarter, 2s. 8d.; per half-year, 5s. 3d.; per annum, 10s. 6d. Foreign:—Per Annum, 15s. 2d.; payable, in all cases, in advance.

### NOTICE TO CORRESPONDENTS.

All MS., Letters, Books for Review, and other matters intended for the Editor should be addressed The Editor, The Lodge, Porchester Square, London, W.

The Editor cannot undertake to return rejected MS., even when accompanied by stamped directed envelope.

**"Burdett's Hospital Annual," 1895,**

In Active Preparation.

Sixth year of publication. About 900 pp. Scarlet cloth, gilt lettered. Price 5s. A most complete guide to British, Colonial, Indian, and American Hospitals, Dispensaries, Nursing and Convalescent Institutions, and Asylums. A few copies of the "Annual" for 1894 may still be obtained on application to the Publishers, The Scientific Press (Limited), 428, Strand, London, W.C.

**NOTICE.—The Index for Vol. XVI. (ending September 24th, 1894) is on sale at the Office of this Journal, Price 2½d., post free.**

### SCRAPS AND GLEANINGS.

Chicago is taking up the question of unhealthy bakeries.

The Dundee Royal Infirmary has recently received altogether £523 11s. 8d. in donations from various local public works.

The annual meeting of the Birmingham Dental Hospital has just been held. The financial position of the institution, although presenting a balance to the bad, showed a decided improvement on that of last year.

The Duke of Devonshire presided at the annual meeting of the Derbyshire Hospital for Sick Children, which has just taken place. His Grace has consented to become president of the institution for the ensuing year.

An evening concert in aid of the funds of the Metropolitan Hospital, Kingsland Road, was held on Thursday in this week at the Town Hall, Shoreditch, the Lord Mayor honouring the entertainment with his patronage and presence.

The annual entertainment to the patients of St. John's Hospital for Diseases of the Skin, Leicester Square, took place last week. It was reported that the income for the year had been £3,645, an increase over the previous twelve months, and that 178 in-patients and 6,306 out-patients had been treated during the twelve months.

The secretary of the Hospital for the Diseases of Women is making an appeal for help to the charitable public. The new building is now several feet above the ground, and the establishment of a maintenance fund to carry on the good work done in the old hospital is urgently needed. By giving £5 to the institution one patient may be sent yearly.

Mr. Albert Boyce, the secretary of the Devon and Exeter Hospital, is appealing for funds, and also for gifts in kind. Help in this latter form is of greater assistance than is always realised. Clothing for the poorer patients, vegetables, &c., would mean little to many of the well-to-do in Exeter, and very much to their neighbours in the hospital wards.

The Christmas treat to the little inmates of the Evelina Hospital for Sick Children, Southwark Bridge Road, proved a great success this year. The wards were tastefully decorated with flowers and ferns and fairy lamps. Each child received presents. There are 60 children at present in the hospital, whilst 808 have been treated as out-patients during the past year.

The inmates of the Deaf and Dumb Children's Home, of which there are four, were entertained last week through the kindness of friends at Bishop Wilson's Memorial Hall, Islington. About 400 of the children assembled. The secretary states that the society is suffering from a financial depression, and to carry on the work an increased income of £300 per annum is required.

Health officers appear to be having hard times in the United States. In several towns petitions have been signed for the removal of the M.O.H., either because he has not enforced quarantine and vaccination to the satisfaction of the inhabitants, or because he has been too severe in so doing. The happy medium seems to be hard of attainment in America.

There was a large attendance this month at the annual meeting of subscribers to St. Catherine's Home for Incurables at Bradford. During the greater part of the past year all the beds have been occupied. The report pointed out that, notwithstanding the strict economy which had been exercised, the year's balance-sheet showed an excess of expenditure over income of £22 3s. 1d.

The annual meeting of the Hospital Saturday and Sunday movement at Wigan shows this year receipts amounting to £260 5s. 9d. The expenses of the extension of the Gidlow Wing to the Wigan Infirmary is being borne by money left for that purpose by Miss Gidlow, which is carrying out the deceased's desire that the institution should meet the needs of the district.

At the meeting of the municipal engineers, recently held at Middlesborough, Mr. Stainthorpe read a paper on "The River Tees Floating Hospital." The building erected on the float consisted of two ward blocks and a large administrative block midway between them. The cost of the hospital was approximately £10,000—£3,000 of this being spent in dredging the berth.

The past inmates of the convalescent homes in Wales established by the Birmingham hospitals took place last month in the Town Hall at Birmingham. The guests numbered about 2,000 persons. A presentation of a writing table was made to the honorary medical officer of the convalescent homes. A concert afterwards took place, followed by an exhibition of limelight views of the homes.

The question as to whether bycicling is healthy or injurious for women has been agitating the medical mind of late. In reply to a request for opinions on the point by Dr. Douglas Hogg, in the "Paris Journal of Medicine," forty-eight answers have been received from physicians of all nationalities. Nine were unfavourable, three recommended the exercise conditionally, while the remaining thirty-six declared in favour of it if practised in moderation.

At the annual meeting of the friends and subscribers of the Mater Misericordiæ Hospital, Dublin, the necessity of the erection of a new wing was unanimously agreed to, and a subscription list was started, the Archbishop of Dublin leading with a donation of £500. Cordial recognition was given to the excellent work done by this institution, and to the zeal and energy shown by the Sisters of Mercy in its administration. The princely subscription of £500 given by his Grace the Archbishop of Dublin, is only part of the service and support which he gives to the new undertaking. The speech made by his Grace in favour of this charity puts its claims before the city and country with irresistible clearness and force.

The movement in favour of instituting public slaughter-houses on the model of those on the continent, where an excellent system of meat control prevails, is gaining ground in England. In Paris, Brussels, and Berlin, the abattoirs are under special veterinary supervision, carried on with the aid of trained inspectors and skilled microscopists. The system cannot be so elaborately practised in small towns, but the appointment of a reliable practical butcher as superintendent, under the medical officer of health, is a step in the right direction. The Model Abattoir Society show evidence that under these circumstances in towns where public abattoirs have been erected, the sale of diseased meat has become almost impossible, and the nuisance arising from insanitary private slaughter-houses is prevented.

### THE STUDENT OF MEDICINE.

### APPOINTMENTS.

T. H. Ashby, M.B.Toronto, L.R.C.P.Lond., appointed Medical Officer to the Workhouse of the Shardlow Union. F. J. Flavin, L.R.C.P., L.R.C.S.Edin., appointed Medical Officer for the No. 7 (Newton and Godley) District of the Ashton-under-Lyne Union. A. Greenwood, L.S.A., appointed Medical Officer for the Haggerston East District of the Parish of St. Leonard, Shoreditch. C. Hodgson, M.R.C.S., L.R.C.P.Lond., appointed House Surgeon to the County Hospital, York. S. H. House, M.B., Ch.B.Vict., appointed Resident Medical Officer to the Grimsby and District Hospital. R. B. James, M.R.C.S., L.R.C.P., appointed Resident Surgeon to the Birmingham General Dispensary. E. W. Lewis, M.R.C.S.Eng., L.R.C.P.Lond., appointed Assistant Anæsthetist to the West London Hospital, Hammersmith. R. W. Marsden, M.B., B.Sc., Ch.B., appointed Resident Officer to the Monsall Fever Hospital. W. Milligan, L.R.C.P. Edin., M.R.C.S.Eng., appointed Medical Officer for the Cumberworth District of the Huddersfield Union. R. W. Nesfield, M.B., Ch.B.Vict., appointed a Resident Medical Officer of St. Mary's Hospital, Manchester. H. J. O'Brien, L.R.C.P., L.R.C.S.I., appointed Medical Officer for the West District of he Poplar Union. C. Planck, jun., M.A.Cantab., L.R.C.P. Lond., M.R.C.S.Eng., late House Surgeon to St. Thomas's Hospital, appointed Junior Assistant Medical Officer to the East Sussex County Asylum, Hayward's Heath. W. A. Propert, M.R.C.S.Eng., L.R.C.P.Lond., appointed House Physician to the West London Hospital.

**THE STUDENT OF MEDICINE—*continued.***

J. R. M. Richmond, L.S.A., appointed Medical Officer of the Overton Penley District of the Ellesmere Union. S. Riseley, M.D.Edin., appointed Senior House Surgeon to the Halifax Infirmary. T. D. S. Shaw, M.B., C.M.Edin., appointed Medical Officer of the North District of the Cirencester Union. G. A. Sutherland, M.A., M.D., M.R.C.P., appointed Physician to Out-patients at Paddington Green Children's Hospital. S. G. Tippett, M.R.C.S., L.R.C.P.Lond., appointed Senior House Physician to the Westminster Hospital. Dr. Tynan, appointed Resident Surgeon to the St. Vincent's Hospital, Dublin. E. S. Yonge, M.B., C.M.Edin., appointed Assistant Medical Officer to the Manchester Hospital for Consumption and Diseases of the Chest.

### VACANCIES.

The following vacancies are announced this week, the amount of salary in each case being given after the name of the institution:

**Resident Medical Officers.**

Bolton Union.—Medical Officer for the Workhouse at Fishpool. Salary £200 per annum and a furnished house. Application (on forms to be obtained of the Clerk) to Simpson Cooper, Clerk to the Guardians, 28, Mawdsley Street, Bolton, by January 14th.

Parochial Board of Barony Parish of Glasgow.—Senior Assistant to the Medical Superintendent of the Barony Parochial Asylum, Woodilee, Lengie, near Glasgow. Salary £200 per annum, with board, apartments, &c. Applications, marked "Woodilee Medical Assistant," to Jas. R. Morton, Acting Inspector of Poor, Barony Parish Chambers, 38, Cochrane Street, Glasgow, by January 12th.

West Herts Infirmary, Hemel Hempstead.—House Surgeon and Dispenser, doubly qualified and unmarried. Appointment for two years. Salary, £100 per annum, with board, furnished rooms, fire, lights, attendance, and washing. Applications to the Secretary by January 17th.

Victoria Hospital for Sick Children, Queen's Road, Chelsea, S.W.—House Physician and House Surgeon for the in-patients. Honorarium, £50 per annum and board and lodging in each case. Applications to the Secretary by January 12th.

**Non-Resident Medical Officers.**

Hospital for Sick Children, Great Ormond Street, W.C.—Two Assistant Physicians; must be F. or M.R.C.P.Lond. Applications to the Secretary by January 14th.

### UNIVERSITIES AND COLLEGES.

UNIVERSITY OF CAMBRIDGE.

Third M.B. Examination: Part II.—Medicine: Bagshawe, B.A., Gonv. and Cai.; Barraclough, B.A., Joh.; Bates, B.A., Queen's; Bond, B.A., non-coll.; Burton, B.A., King's; A. V. Clarke, B.A., Gonv. and Cai.; Cotter, B.A., Trin.; Cowan, B.A., King's; E. C. Daniel, B.A., Emm.; Falkener, M.A., King's; G. H. Field, B.A., Cla.; Fison, B.A., Corp. Chr.; H. C. Goodman, B.A., Joh.; Gordon, B.A., King's; L. N. Harding, B.A., H. Selw.; Heaton, B.A., Trin.; Hedges, B.A., Sid. Suss.; Henderson, B.A., Joh.; Irving, B.A., Gonv. and Cai.; Key, B.A., Emm.; T. P. King, B.A., Joh.; Marks, M.A., Jes.; H. Marshall, B.A., Gonv. and Cai.; G. P. Mathew, B.A., Trin. H.; Milward, B.A., Cla.; Moysey, B.A., Gonv. and Cai.; Nachbar, M.A., Cla.; W. Norbury, B.A., Trin.; Norris, M.A., Chr.; Paget, Gonv. and Cai.; Pead, B.A., Down; Ll. C. P. Phillips, B.A., Gonv. and Cai.; Ll. Powell, B.A., Trin.; Powell, B.A., Cla.; Sandall, B.A., Joh.; Sheppard, B.A., Chr.; Smith, B.A., Trin.; Taylor, B.A., Emm.; Thomas, B.A., Chr.; Whichello, B.A., Sid. Suss.; White, B.A., Sid. Suss.; Wingfield, M.A., Gonv. and Cai.

ROYAL COLLEGE OF PHYSICIANS IN IRELAND.

The following registered medical practitioner has been admitted a Licentiate in Medicine: P. Merrin, L.R.C.S.I., L.A.H.

The undermentioned candidate has been admitted a Licentiate in Medicine and Midwifery, under the Conjoint Scheme with the Royal College of Surgeons in Ireland: E. J. Tynan.

UNIVERSITY OF EDINBURGH.

The University and extramural classes resume after the Christmas recess of seventeen days on January 8th.

A meeting of the University Court will be held on January 14th, for the appointment of Examiners in Medicine and in Science, and for other competent business.

At its last meeting the Court appointed Dr. J. C. Affleck additional Examiner in Medicine in its bearings on public health. The course in Chemistry, given by Mr. T. W. Drinkwater, Ph.D., and the course in Zoology, by Mr. T. Arthur Thomson, M.A., were recognised as qualifying women students for graduation in Arts.

UNIVERSITY OF LONDON.

B.S. Examination (Honours).—Surgery—First Class: J. H. Fisher (scholarship and gold medal), St. Thomas's Hospital; H. Davies (gold medal), Guy's Hospital; *C. S. Wallace, St. Thomas's Hospital; S. W. F. Richardson, B.Sc., St. Thomas's Hospital. Second Class: W. R. Smith, King's College; G. H. Cowen, London Hospital. *Obtained the number of marks qualifying for a gold medal.

UNIVERSITY OF GLASGOW.

Graduation Ceremony.—At the University on November 8th degrees were conferred in Arts, Science, Divinity, Law, and Medicine. Of the medical graduates, seven received the higher degree of M.D.; one of these, Dr. W. P. Jack, the son of the Professor of Mathematics in the University, received the degree with honours, for a thesis on "Observations on the Analysis of Voluntary Muscular Movements by Certain New Instruments." The degrees of Bachelor of Medicine and Master in Surgery were conferred on twenty-two, of whom two were ladies, students of Queen Margaret College.

